# Sand Fly Fauna (Diptera: Psychodidae): Association Between Climatic Variables and Natural *Leishmania* Infection in Araçatuba, Brazil

**DOI:** 10.3390/microorganisms14020500

**Published:** 2026-02-20

**Authors:** Graziella Borges Alves, Debora Regina Romualdo da Silva, Elis Domingos Ferrari, Lilian Aparecida Colebrusco Rodas, Alex Akira Nakamura, Carolina Beatriz Baptista, Camila Pedrozo Rodrigues Furlan, Keuryn Alessandra Mira Luz Requena, Gabriele Zaine Teixeira Debortoli, Thais Rabelo Santos-Doni, Katia Denise Saraiva Bresciani

**Affiliations:** 1 School of Veterinary Medicine, São Paulo State University (Unesp), Araçatuba 16050-680, SP, Brazil; graziella.b.alves@unesp.br (G.B.A.); debora.romualdo@unesp.br (D.R.R.d.S.); alex.nakamura@unesp.br (A.A.N.); carolina.beatriz@unesp.br (C.B.B.); camila.furlan@unesp.br (C.P.R.F.); kaml.requena@unesp.br (K.A.M.L.R.); katia.bresciani@unesp.br (K.D.S.B.); 2 Union of Colleges of the Great Lakes (UNILAGO), São José do Rio Preto 15030-070, SP, Brazil; elisd.ferrari@yahoo.com.br; 3 Pasteur Institute, Center for Disease Control, State Department of Health, São Paulo 05508-020, SP, Brazil; colerodas@gmail.com; 4 Institute of Agricultural Sciences (ICA), Federal University of Jequitinhonha and Mucuri Valleys (UFVJM), Avenida Universitários, 1000, Unaí 39100-000, MG, Brazil; gabriele.zaine@ufvjm.edu.br

**Keywords:** Phlebotominae ecology, vector-borne diseases, entomological surveillance, seasonal dynamics, canine visceral leishmaniasis

## Abstract

Visceral leishmaniasis (VL) is a zoonosis of major public health importance. In urban areas, *Lutzomyia longipalpis* is the primary vector of *Leishmania* (L.) *infantum*. This study assessed the seasonality, spatiotemporal distribution, and climatic factors associated with *L. longipalpis* abundance in Araçatuba, São Paulo State, and detected *Leishmania* spp. DNA in captured females. Monthly collections were conducted from March 2023 to February 2024 in 72 households across eight urban areas using CDC-type light traps placed indoors and in peridomestic environments. A total of 1641 specimens (1516 males and 125 females) were captured, with 92.4% originating from peridomestic areas. Area 3 had the highest density (*n* = 671) and was the only area with PCR-positive females (*n* = 3). Vector activity peaked in December 2023 (*n* = 335). Male abundance differed significantly among peridomestic areas, particularly between Areas 3, 5, 6, and 7. In peridomestic areas, higher final temperatures increased vector abundance, whereas higher initial temperatures and humidity reduced it. Indoors, final temperature, humidity, and month were significant predictors. *L. longipalpis* exhibited a defined seasonal and spatial pattern influenced by climatic conditions. The detection of PCR-positive females (Area 3) highlights the epidemiological role of the vector and underscores the need for targeted interventions to control VL.

## 1. Introduction

Visceral leishmaniasis (VL) is a parasitic zoonosis with a wide geographic distribution and is considered one of the most globally significant neglected tropical diseases. It is estimated that over 50,000 cases are reported annually, particularly in regions of Asia, Africa, and Latin America, where socioeconomic, environmental, and ecological factors contribute to the persistence and expansion of the disease [1].

In Brazil, VL is endemic in several regions and represents a serious public health concern, with increasing records of geographic expansion and urbanization of transmission, especially in recent decades [2,3].

The etiological agent of the visceral form of the disease in Brazil is *Leishmania infantum* (Kinetoplastida: Trypanosomatidae), primarily transmitted through the bite of female sand flies of the species *Lutzomyia longipalpis* (Diptera: Psychodidae: Phlebotominae), which is the main confirmed vector in most urban transmission foci [4,5,6].

The spread of VL to areas previously considered disease-free has been linked to environmental changes, migratory flows, population density, and the presence of infected dogs, which are the primary urban reservoirs [3].

In the state of São Paulo (SP), the municipality of Araçatuba represents a significant milestone in the history of visceral leishmaniasis. In 1998, the first cases of canine VL were recorded, followed in 1999 by the first reports of autochthonous human cases [7,8]. Since then, both the municipality and its surrounding areas have become endemic for the disease, with sustained transmission and continued impact on local public health. The municipality and surrounding areas have remained a relevant focus for surveillance, with integrated control efforts including entomological monitoring, environmental management, and canine reservoir surveillance. In this context, identifying seasonal peaks and spatial hotspots of vector abundance and infection may support more efficient, geographically targeted interventions [9].

This study characterized the sand fly fauna in Araçatuba, Brazil, analyzed the association with climatic variables, and evaluated natural *Leishmania* infection to support strategies for visceral leishmaniasis prevention and control in endemic and expanding areas.

## 2. Materials and Methods

All experimental procedures were carried out in full accordance with ethical standards and approved by the Ethics Committee on Animal Use (CEUA) of the Faculdade de Odontologia de Araçatuba and the Faculdade de Medicina Veterinária de Araçatuba (FMVA), UNESP, Campus de Araçatuba (protocol FOA n° 00870-2016).

The research was carried out in the urban area of Araçatuba (21°12′32″ S, 50°25′58″ W), located at an altitude of 390 m, in the state of São Paulo, southeastern Brazil. According to data from the Brazilian Institute of Geography and Statistics (IBGE, 2024), the municipality has an estimated population of 207,775 inhabitants (https://ftp.ibge.gov.br/Estimativas_de_Populacao/Estimativas_2024/estimativa_dou_2024.pdf (accessed on 23 September 2025)) and occupies an area of 1167.126 km^2^ (https://www.ibge.gov.br/cidades-e-estados/sp/aracatuba.html (accessed on 23 September 2025)). Araçatuba lies within the Atlantic Forest biome and is characterized by a tropical Aw climate under the Köppen–Geiger classification. The mean annual temperature is 24.1 °C, and the average precipitation reaches 1328 mm, with the rainy season concentrated in the summer months (https://pt.climate-data.org/america-do-sul/brasil/sao-paulo/aracatuba-4222/ (accessed on 23 September 2025)).

The spatial division of the municipality of Araçatuba, São Paulo, Brazil, adopted in this study was based on the urban census sectors defined by the Brazilian Institute of Geography and Statistics (IBGE) for the 2022 demographic census, corresponding to the main district. The numbered Areas (1–8) correspond to groups of contiguous census sectors, stratified according to geographical dispersion criteria and environmental characteristics. These census-based spatial units were employed for the analysis of sand fly captures in peridomestic and intradomestic environments and *Leishmania* PCR positivity, allowing comparisons among subregions with distinct urbanization and environmental profiles.

Two trapping cages were positioned approximately 50 cm above the ground in each household between March 2023 and February 2024. The traps were exposed for 12 h intervals, from 7:00 p.m. to 7:00 a.m., and remained in situ for a total of 72 h. In addition, entomological surveys were performed using seven standard CDC light traps, with their bulbs removed before deployment. These traps were placed at two distinct locations: one inside the residence, typically in the main bedroom, and another near the dog’s sleeping area or at the entrance of the kennel. Thus, each trap was positioned in proximity to both human and canine hosts. Collected sand flies were counted and sexed under a stereomicroscope. Following the procedure described by Gaglio et al. [10], the specimens were prepared for microscopic examination and identified to the species level using morphological criteria. As previously noted, *Lutzomyia longipalpis* females were subjected to the PCR protocol described above, following sexual differentiation and morphological confirmation.

We performed nested SSU rRNA-PCR using the primer sets and conditions previously described by [11,12,13], including negative controls in all runs. The amplified products were analyzed by electrophoresis on a 2.0% agarose gel containing ethidium bromide, and 10 µL of each PCR product was compared against a 100 bp DNA ladder. DNA bands were visualized under ultraviolet transillumination.

Statistical analyses were conducted using STATA/SE version 16.1 (StataCorp LLC, College Station, TX, USA) and Microsoft^®^ Excel^®^ from the Microsoft 365 package. The threshold for statistical significance was established at *p* < 0.05.

To evaluate differences in *L. longipalpis* abundance among the eight urban areas studied, the data were first assessed for normality and homoscedasticity. As the dataset did not meet the assumptions for parametric testing, nonparametric methods were applied. Differences in sand fly counts among areas (peridomestic and intradomestic) were tested using the Kruskal–Wallis test, followed by Dunn’s post hoc multiple comparisons with Bonferroni correction when applicable.

Temporal variation in sand fly abundance (monthly comparisons) and the influence of microclimatic variables (initial and final temperature, initial and final humidity) were assessed using Generalized Linear Models (GLMs). Given the distributional characteristics of the data and the presence of overdispersion, models were fitted using a square root link function, appropriate for count datasets with heterogeneous variance.

For each GLM, Likelihood Ratio Tests (LRTs) were used to determine the statistical significance of individual predictors. In the peridomestic models, the explanatory variables tested included initial temperature, final temperature, initial humidity, final humidity, and month.

Similarly, intradomestic models evaluated the same climatic variables and monthly variation. When significant monthly effects were detected, pairwise post hoc contrasts with Bonferroni adjustment were performed to identify specific differences between months.

Spatial distribution analyses were conducted using QGIS^®^ software version 3.26 (Buenos Aires). Kernel density estimation and buffer zone (200 m and 800 m) evaluations were performed separately and were not part of the inferential statistical models.

## 3. Results

In Araçatuba, a total of 1226 *L. longipalpis* were collected, including 1148 from peridomestic areas (87.4% males; 1003/145 males/females) and 78 from intradomestic areas (80.8% males; 63/15 males/females) (Table 1). Peridomestic abundance was markedly higher in Area 3 (*n* = 671; 622 males and 49 females), followed by Area 6 (*n* = 201) and Area 2 (*n* = 92), while Area 8 had the fewest specimens (*n* = 2). Intradomestically, the highest total was recorded in Area 4 (*n* = 37), with much lower values elsewhere (Table 1).

Over the study period, peridomestic captures peaked in December 2023 (*n* = 335), and the indoor maximum occurred in August 2023 (*n* = 19), with a small rise in females in February 2024 (*n* = 4). Microclimatic conditions at the capture sites showed little variation among areas: initial temperature ranged from 26.9 to 28.0 °C, final temperature from 21.2 to 21.9 °C, initial humidity from 46.0% to 51.9%, and final humidity from 56.7% to 59.5%.

**Figure 1:**
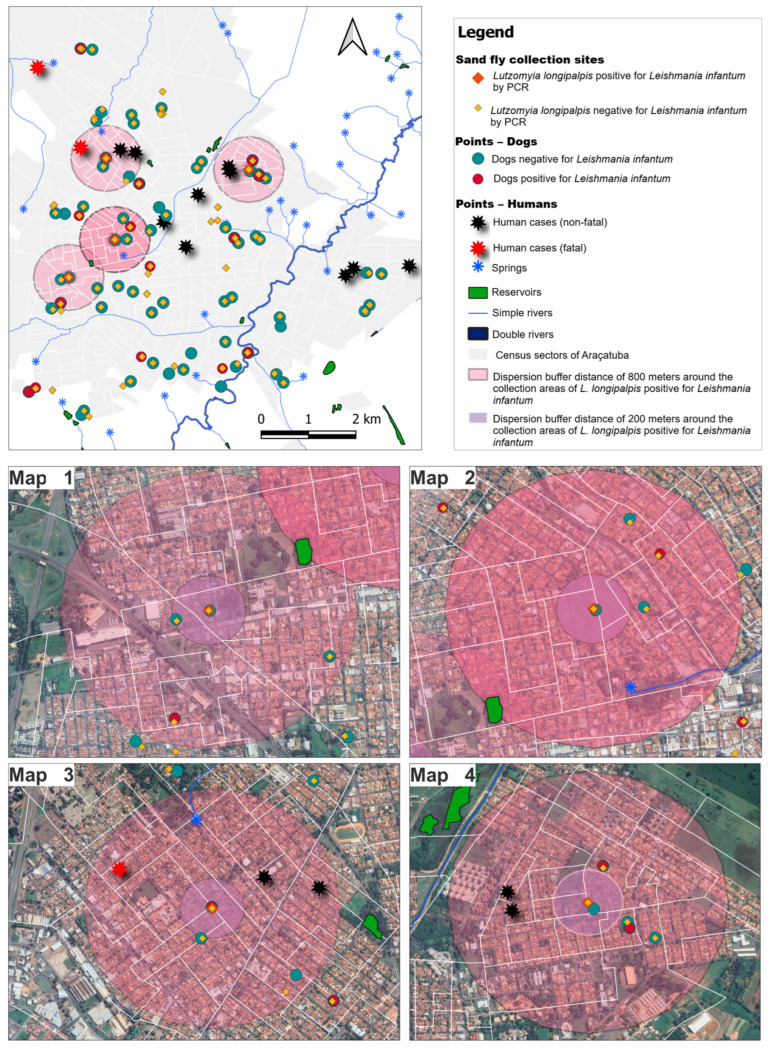
Map showing buffer zones (200–800 m) around *Lutzomyia longipalpis* collection sites by *Leishmania infantum* presence (PCR).

Kernel density maps showed multiple hotspots of *L. longipalpis* abundance in central–eastern urban sectors (Figure 1).

**Figure 2:**
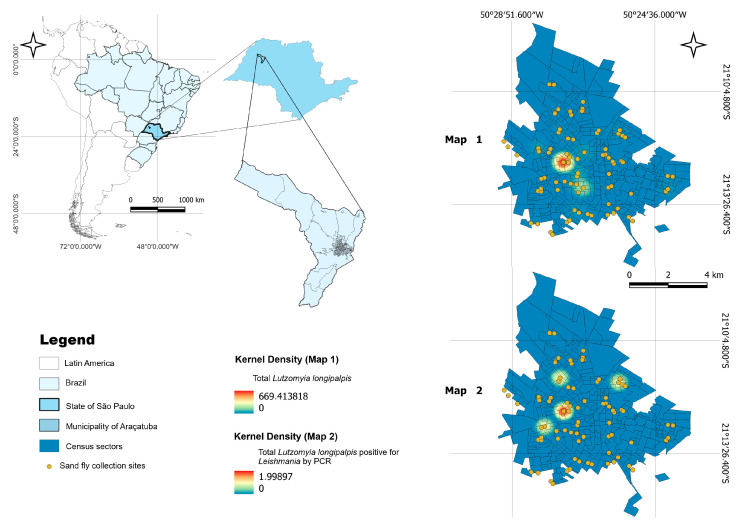
Spatial distribution and density of *Lutzomyia longipalpis* and specimens positive for *Leishmania* by PCR in the municipality of Araçatuba, São Paulo, Brazil.

A second kernel surface based on PCR-positive *L. longipalpis* indicated smaller and more focal intensity peaks (0–1.999). Buffer analyses centered on positive vector sites (200 m and 800 m) revealed co-location of canine infections and human VL cases, including fatal cases, within or adjacent to these buffers, especially in four clusters displayed in the high-resolution panels (Figure 2). Hydrological features, including springs, small streams, and drainage channels, intersected several of these clusters.

Nonparametric comparisons revealed heterogeneity among peridomestic sites for male counts (*p* < 0.01), but not for females (*p* = 0.141). Dunn–Bonferroni post hoc analysis identified significant differences between Areas 3 and 5, 6 and 5, 3 and 7, and 6 and 7 (all *p* < 0.01). Intradomestically, male counts differed between Area 4 and Areas 2 and 7 (*p* < 0.05), while indoor female counts did not differ among areas (Table 2).

Peridomestic activity showed a clear summer peak, with the highest monthly total in December 2023 (*n* = 335) and the lowest in September 2023 (*n* = 8). In intradomestic settings, captures peaked in August 2023 (*n* = 19) and dropped to zero in September 2023. Overall, peridomestic captures ranged from 40 to 335, and indoor captures ranged from 0 to 19 throughout the study period (March 2023–February 2024). Detailed monthly totals by sex are presented in Table 3.

Post hoc GLM comparisons revealed significant temporal variation in sand fly abundance across months. In peridomestic environments, December 2023 showed markedly higher counts than most other months (all *p* < 0.001), including April, May, July, August, and November, confirming a strong seasonal peak at the end of the year. In contrast, June and October 2023 presented significantly lower counts compared with April 2023 (*p* < 0.05).

In intradomestic areas, significant differences were observed mainly between August 2023 and other months, particularly December 2023, March 2023, May 2023, and November 2023 (*p* < 0.05). These results, summarized in Table 4, indicate clear seasonal fluctuations in both peridomestic and intradomestic sand fly activity, with maximum abundance concentrated in the warmer, more humid summer months.

In intradomestic GLMs, likelihood ratio tests indicated that final temperature (χ^2^ = 3.93, df = 1, *p* = 0.047) and final humidity (χ^2^ = 3.02, df = 1, *p* = 0.082) were the only variables influencing sand fly counts, with positive and negative effects, respectively. Month effects were also significant (χ^2^ = 34.38, df = 11, *p* < 0.001), confirming temporal variation in indoor captures. Model coefficients revealed a positive association between final temperature (Estimate = +0.01287, SE = 0.0058, z = 2.22, *p* = 0.027) and phlebotomine abundance. In contrast, final humidity showed a slight negative relationship (Estimate = −0.00377, SE = 0.00184, z = −2.05, *p* = 0.041) (Table 5).

Consistently, post hoc contrasts demonstrated that August 2023 had significantly higher indoor counts than March, May, October, November, and December 2023 (*p* < 0.05 for all) (Table 4). These results suggest that intradomestic sand fly activity increased under warmer and drier conditions, reaching its highest levels in late winter (August).

## 4. Discussion

The analysis of seasonality, spatiotemporal distribution, and climatic factors associated with the abundance of *L. longipalpis* in Araçatuba reveals an epidemiological pattern consistent with findings reported in other endemic regions of Brazil while also highlighting local specificities that may influence surveillance and control strategies. The peak of vector activity recorded in the peridomestic area in December 2023 confirms the strong influence of seasonality on the species’ population dynamics, a finding widely documented in endemic areas such as Dracena (SP), where eight years of monitoring identified peridomestic predominance and a population increase at the beginning of the rainy season [14,15].

In Campo Grande, Mato Grosso do Sul (MS), Brazil, vector activity remained constant throughout the year but exhibited marked peaks immediately after periods of heavy rainfall [16], a phenomenon also observed in Pernambuco [17], reinforcing that seasonality responds to microclimatic variations that directly affect the survival, reproduction, and dispersal of the species [18].

In the present study, statistical modeling (GLM) revealed a positive association between higher final temperatures and sand fly abundance in the peridomestic area, while higher initial temperatures and humidity exerted a significant negative effect. These findings align with the observations of [19], which identified a positive correlation between the abundance of *L. longipalpis* and increases in temperature and accumulated precipitation, as well as variations in humidity, suggesting that daily oscillations in environmental parameters may modulate the vector’s nocturnal activity and capture rates.

In urban settings, studies have shown that population dynamics may also be influenced by structural and socioeconomic factors, including irregular solid waste disposal, forest fragments, and water drainage, which directly affect the availability of shelters and food sources [20]. Although climatic factors explain a substantial portion of temporal variation, anthropogenic aspects must also be incorporated into the interpretation of spatial patterns. In contrast to the present findings, Garcia et al. [21] did not identify a correlation between phlebotomine abundance and climatic factors such as temperature and precipitation.

The heterogeneous distribution of sand flies among the study areas, with a marked concentration in Area 3—the only site with PCR-positive females for *Leishmania* spp.—reinforces the presence of microhabitats favorable to the maintenance of the vector cycle. Studies conducted in Argentina by Santini et al. [22] demonstrated that macrohabitat variables, such as vegetation cover and proximity to water bodies, are associated with vector presence, while abundance is more closely related to microenvironmental conditions, including shading, the presence of animals, and the accumulation of organic matter. In Araçatuba, the spatial overlap between capture points of infected females, human and canine cases, and hydrological features suggests the coexistence of environmental factors and domestic reservoirs that favor transmission.

The predominance of captures in the peridomestic area observed in this study is consistent with the literature and has relevant epidemiological implications. Chicken coops, although not competent reservoirs for *Leishmania*, have been described as structures that increase the attraction and maintenance of sand fly populations in domestic areas, indirectly contributing to the risk of transmission [23,24,25].

The detection of infected females at only one focal point underscores the importance of targeted surveillance strategies that prioritize areas with higher vector density and confirmed risk, thereby optimizing control resources. Moreover, the correlation between areas of greater vector abundance and the presence of infected dogs reinforces the role of the canine reservoir in maintaining urban transmission [26,27,28] [29]. Continuous surveillance, coupled with the incorporation of predictive climatic variables, can enhance the early detection of high-risk periods and guide preventive interventions.

This study has some limitations. The one-year sampling period precluded the assessment of interannual variability. Environmental covariates were not incorporated into the statistical models, and spatial analyses were exploratory. Additionally, the low number of infected females prevented robust modeling of infection predictors.

In summary, the results from Araçatuba reaffirm that the ecology of *L. longipalpis* is strongly modulated by seasonal and microenvironmental factors, yet also dependent on anthropogenic determinants that shape the vector’s spatial distribution. The convergence with findings from other endemic regions strengthens the external validity of the observed patterns and supports the recommendation of targeted interventions synchronized with seasonal dynamics, integrating entomological surveillance, reservoir control, and environmental improvements as pillars of sustainable urban visceral leishmaniasis management.

## 5. Conclusions

The results from Araçatuba demonstrate that *Lutzomyia longipalpis* abundance is strongly influenced by seasonality and climatic variation, with marked population peaks during the rainy months and a heterogeneous spatial distribution characterized by well-defined hotspots. The detection of *Leishmania* spp.-infected females restricted to a specific area highlights the focal nature of transmission risk within the urban environment. These findings emphasize the relevance of spatiotemporal heterogeneity for vector surveillance and support the use of targeted, seasonally oriented control measures in urban areas endemic for visceral leishmaniasis.

## Figures and Tables

**Table 1:** Absolute frequency of *Lutzomyia longipalpis* by capture location, in the peridomestic and intradomestic areas, in the urban area of Araçatuba, SP, Brazil, from March 2023 to February 2024.

Area	Peridomestic				Intradomestic				Positive PCR—*Leishmania*			
	Total	CI 95% *			Total	CI 95% *			Total	CI 95% *		
Female												
1	7.0	1.9	-	12.1	3.00	−0.4	-	6.4	1.0	1.0	-	3.0
2	10.0	2.7	-	17.3	1.00	−1.0	-	3.0	1.0	1.0	-	3.0
3	49.0	20.7	-	77.3	1.00	−1.0	-	3.0	3.0	0.4	-	6.4
4	15.0	0.8	-	29.2	3.00	−0.4	-	6.4	0.0	NC		
5	11.0	0.1	-	21.9	1.00	−1.0	-	3.0	0.0	NC		
6	48.0	11.5	-	84.5	3.00	−0.4	-	6.4	0.0	NC		
7	4.0	0.1	-	7.9	3.00	−0.4	-	6.4	0.0	NC		
8	1.0	−1.0	-	3.0	0.00	NC			0.0	NC		
Total	145.0				15.0				5.0			
Male												
1	40.0	6.1	-	73.9	3.0	−0.4	-	6.4	NC			
2	82.0	27.0	-	137.0	3.0	−1.4	-	7.4				
3	622.0	229.5	-	1014.5	11.0	0.9	-	21.1				
4	64.0	16.7	-	111.3	34.0	1.1	-	66.9				
5	26.0	−2.7	-	54.7	5.0	0.6	-	9.4				
6	153.0	75.0	-	231.0	5.0	−1.5	-	11.5				
7	15.0	5.3	-	24.7	2.0	−0.8	-	4.8				
8	1.0	−1.0	-	3.0	0.0	NC						
Total	1003.0				63.0							
Total												
1	47.0	12.6	-	81.4	6.0	1.2	-	10.8	NC			
2	92.0	33.8	-	150.2	4.0	−0.8	-	8.8				
3	671.0	255.2	-	1086.8	12.0	1.7	-	22.3				
4	79.0	24.9	-	133.1	37.0	3.9	-	70.1				
5	37.0	−0.4	-	74.4	6.0	1.2	-	10.8				
6	201.0	99.2	-	302.8	8.0	0.7	-	15.3				
7	19.0	7.9	-	30.1	5.0	0.6	-	9.4				
8	2.0	−1.9	-	5.9	0.0	NC						
Total	1148.0				78.0							

*: Confidence interval.

**Table 2:** Multiple comparisons between different areas in the distribution of phlebotomine sandflies, in peridomestic and intradomestic areas, in the urban area of Araçatuba, SP, Brazil, during the period from March 2023 to February 2024.

Area	Peridomestic			Area	Intradomestic		
	*p* ^1^	Z-Statistic	*p* Adjusted ^2^		*p* ^1^	Z-Statistic	*p* Adjusted ^2^
♀ ^3^							
	0.1414				0.8228		
♂ ^4^							
Area 3 vs. Area 5	0.0001	4.0105	0.0008	Area 4 vs. Area 2	0.0285	−3.0773	0.0292
Area 6 vs. Area 5		−4.0095	0.0009	Area 4 vs. Area 7		3.1305	0.0244
Area 3 vs. Area 7		3.5778	0.0049				
Area 6 vs. Area 7		3.5721	0.0050				
Total							
Area 3 vs. Area 7	0.0031	3.1041	0.0267		0.1341		
Area 6 vs. Area 7		3.0999	0.0271				

^1^: Kruskal–Wallis; ^2^: Dunn’s test with Bonferroni adjustment; ^3^: Female; ^4^: Male.

**Table 3:** Number of *Lutzomyia longipalpis* specimens, by sex (male and female) and trap location (peridomestic and intradomestic) over several months, in the urban area of Araçatuba, SP, Brazil, from March 2023 to February 2024.

Period	Trap Location													
	Peridomestic							Intradomestic						
	♂ ^1^	♀ ^2^	T ^3^	Med ^4^	SD ^5^	Min ^6^	Max ^7^	♂ ^1^	♀ ^2^	T ^3^	Med ^4^	SD ^5^	Min ^6^	Max ^7^
March 2023	104	8	112	0.6	3.8	0	41	7	0	7	0.0	0.3	0	3
April 2023	77	13	90	0.4	2.0	0	23	5	2	7	0.0	0.2	0	2
May 2023	71	6	77	0.4	3.4	0	36	0	1	1	0.0	0.1	0	1
June 2023	31	9	40	0.2	1.2	0	13	9	3	12	0.1	0.4	0	4
July 2023	65	6	71	0.4	3.9	0	49	7	2	9	0.1	0.3	0	2
August 2023	33	12	45	0.2	1.0	0	10	19	0	19	0.1	1.1	0	15
September 2023	5	3	8	0.1	0.8	0	6	0	0	0	0.0	0.0	0	0
October 2023	52	10	62	0.3	2.3	0	19	1	0	1	0.0	0.1	0	1
November 2023	53	8	61	0.4	2.5	0	23	2	0	2	0.0	0.1	0	1
December 2023	292	43	335	1.7	12.9	0	164	1	2	3	0.0	0.1	0	1
January 2024	107	12	119	0.6	3.9	0	42	7	1	8	0.0	0.4	0	6
February 2024	113	15	128	0.6	5.0	0	60	5	4	9	0.0	0.2	0	1

^1^: Male; ^2^: Female; ^3^: Total; ^4^: Mean; ^5^: Standard Deviation; ^6^: Minimum; ^7^: Maximum.

**Table 4:** Post hoc comparisons (GLM) for the different months, in the peridomestic area, using Bonferroni correction, in the urban area of Araçatuba, SP, Brazil, from March 2023 to February 2024.

Compared Variable (Months)	Estimated Difference	Standard Error	Z-Value	Bonferroni Adjusted *p*-Value
**Peridomestic**				
December 2023 vs. April 2023	−0.5476	0.061	−89,815	<0.001
June 2023 vs. April 2023	0.2104	0.0523	40,213	0.044
August 2023 vs. December 2023	−0.6707	0.0636	−105,538	<0.001
August 2023 vs. July 2023	−0.21973	0.0621	−353,851	0.027
August 2023 vs. February 2024	0.49217	0.0572	861,178	<0.001
December 2023 vs. January 2024	0.59589	0.0528	1,129,477	<0.001
December 2023 vs. July 2023	0.45099	0.0855	527,402	<0.001
December 2023 vs. June 2023	0.75803	0.0681	1,112,457	<0.001
December 2023 vs. May 2023	0.58865	0.0672	875,582	<0.001
December 2023 vs. March 2023	0.59615	0.0616	967,412	<0.001
December 2023 vs. November 2023	0.57194	0.0628	910,794	<0.001
December 2023 vs. October 2023	0.69515	0.0611	1,137,613	<0.001
December 2023 vs. September 2023	0.70248	0.1028	683,251	<0.001
February 2024 vs. June 2023	0.26586	0.0656	405,486	0.003
February 2024 vs. October 2023	0.20298	0.0546	372,059	0.013
February 2024 vs. June 2023	0.30703	0.0625	491,018	<0.001
**Intradomestic**				
August 2023 vs. December 2023	0.22933	0.0652	3.515	0.029
August 2023 vs. May 2023	0.25223	0.0527	4.785	<0.001
August 2023 vs. March 2023	0.23823	0.0697	3.420	0.041
August 2023 vs. November 2023	0.23302	0.0647	3.599	0.021
August 2023 vs. October 2023	0.25458	0.0598	4.260	0.001

**Table 5:** Variations in sandfly counts, analyzed using a Generalized Linear Model (Square Root Link Function), in peridomestic and intradomestic areas of Araçatuba, SP, Brazil, from March 2023 to February 2024.

Variables/Area	Likelihood Ratio Tests			Generalized Linear Model (GLM)			
	X ^2^	Df ^1^	*p* ^2^	Estimate	SE ^3^	Z ^4^	*p* ^2^
**Peridomestic**							
Initial temperature	10.82	1	0.001	−0.0228	0.0070	−3.232	0.001
Final temperature	49.37	1	<0.001	0.0430	0.0058	7.460	<0.001
Initial humidity	13.37	1	<0.001	−0.0080	0.0020	−3.999	<0.001
Final humidity	1.15	1	0.284	0.0021	0.0018	1.127	0.260
Months	236.84	11	<0.001				
August 2023–April 2023				−0.1231	0.0529	−2.326	0.020
December 2023–April 2023				0.5476	0.0610	8.982	<0.001
February 2024–April 2023				0.0554	0.0573	0.968	0.333
January 2024–April 2023				−0.0483	0.0606	−0.797	0.425
July 2023–April 2023				0.0966	0.0683	1.414	0.157
June 2023–April 2023				−0.2104	0.0523	−4.021	<0.001
May 2023–April 2023				−0.0411	0.0517	−0.794	0.427
March 2023–April 2023				−0.0486	0.0582	−0.835	0.404
November 2023–April 2023				−0.0243	0.0581	−0.419	0.675
October 2023–April 2023				−0.1476	0.0527	−2.802	0.005
September 2023–April 2023				−0.1549	0.0868	−1.785	0.074
**Intradomestic**							
Initial temperature	0.113	1	0.737	−0.0033	0.00709	−0.465	0.642
Final temperature	3.932	1	0.047	0.01287	0.0058	2.217	0.027
Initial humidity	0.528	1	0.467	9.39 × 10^−4^	0.00215	0.436	0.663
Final humidity	3.017	1	0.082	−0.00377	0.00184	−2.051	0.04
Months	34.377	11	<0.001				
August 2023–April 2023				0.16995	0.05535	3.070	0.002
December 2023–April 2023				−0.05938	0.06135	−0.968	0.333
February 2024–April 2023				0.02396	0.05832	0.411	0.681
January 2024–April 2023				−0.01723	0.06102	−0.282	0.778
July 2023–April 2023				0.05931	0.0707	0.839	0.402
June 2023–April 2023				0.0551	0.05333	1.033	0.301
May 2023–April 2023				−0.08227	0.05413	−1.520	0.128
March 2023–April 2023				−0.06828	0.05849	−1.167	0.243
November 2023–April 2023				−0.06306	0.06026	−1.046	0.295
October 2023–April 2023				−0.08462	0.05558	−1.522	0.128
September 2023–April 2023				−0.02827	0.09073	−0.312	0.755

^1^: degrees of freedom; ^2^: *p*-value; ^3^: standard error; ^4^: Wald z-statistic.

## Data Availability

The raw data supporting the conclusions of this article will be made available by the authors on request.
